# A Potential Serum *N*-glycan Biomarker for Hepatitis C Virus-Related Early-Stage Hepatocellular Carcinoma with Liver Cirrhosis

**DOI:** 10.3390/ijms21238913

**Published:** 2020-11-24

**Authors:** Mikito Higashi, Takeshi Yoshimura, Noriyoshi Usui, Yuichiro Kano, Akihiro Deguchi, Kazuhiro Tanabe, Youichi Uchimura, Shigeki Kuriyama, Yasuyuki Suzuki, Tsutomu Masaki, Kazuhiro Ikenaka

**Affiliations:** 1 Division of Neurobiology and Bioinformatics, National Institute for Physiological Sciences, National Institutes of Natural Sciences, Okazaki, Aichi 444-8787, Japan; mikito.higashi@gmail.com (M.H.); ykano@nips.ac.jp (Y.K.); 2 Mitsubishi Chemical Group Science and Technology Research Center, Yokohama, Kanagawa 227-8502, Japan; tanabe.kazuhiro@mp.medience.co.jp; 3 Department of Physiological Sciences, School of Life Sciences, SOKENDAI (The Graduate University for Advanced Studies), Hayama, Kanagawa 240-0193, Japan; 4 Department of Child Development and Molecular Brain Science, United Graduate School of Child Development, Osaka University, Suita, Osaka 565-0871, Japan; 5 Department of Neuroscience and Cell Biology, Graduate School of Medicine, Osaka University, Suita, Osaka 565-0871, Japan; usui@anat1.med.osaka-u.ac.jp; 6 Addiction Research Unit, Osaka Psychiatric Research Center, Osaka Psychiatric Medical Center, Osaka 541-8567, Japan; 7 Department of Gastroenterology and Neurology, Faculty of Medicine, Kagawa University, Kita-gun, Kagawa 761-0793, Japan; dboy.aki0829haru1018@gmail.com (A.D.); tmasaki@med.kagawa-u.ac.jp (T.M.); 8 Department of Gastroenterological Surgery, Faculty of Medicine, Kagawa University, Kita-gun, Kagawa 761-0793, Japan; szk@med.kagawa-u.ac.jp

**Keywords:** *N*-glycan, serum, biomarker, hepatocellular carcinoma, hepatitis C virus, cirrhosis

## Abstract

Detection of early-stage hepatocellular carcinoma (HCC) is beneficial for prolonging patient survival. However, the serum markers currently used show limited ability to identify early-stage HCC. In this study, we explored human serum *N*-glycans as sensitive markers to diagnose HCC in patients with cirrhosis. Using a simplified fluorescence-labeled *N*-glycan preparation method, we examined non-sialylated and sialylated *N*-glycan profiles from 71 healthy controls and 111 patients with hepatitis and/or liver cirrhosis (LC) with or without HCC. We found that the level of serum *N*-glycan A2G1(6)FB, a biantennary *N*-glycan containing core fucose and bisecting GlcNAc residues, was significantly higher in hepatitis C virus (HCV)-infected cirrhotic patients with HCC than in those without HCC. In addition, A2G1(6)FB was detectable in HCV-infected patients with early-stage HCC and could be a more accurate marker than alpha-fetoprotein (AFP) or protein induced by vitamin K absence or antagonists-II (PIVKA-II). Moreover, there was no apparent correlation between the levels of A2G1(6)FB and those of AFP or PIVKA-II. Thus, simultaneous use of A2G1(6)FB and traditional biomarkers could improve the accuracy of HCC diagnosis in HCV-infected patients with LC, suggesting that A2G1(6)FB may be a reliable biomarker for early-stage HCC patients.

## 1. Introduction

Liver cancer is the fourth leading cause of cancer-related death worldwide [1]. Hepatocellular carcinoma (HCC) is the most common type of primary liver cancer. A high proportion of hepatitis B virus (HBV) and hepatitis C virus (HCV) carriers develop liver cirrhosis (LC) and HCC [2]. Therefore, cirrhotic patients require close surveillance using serum markers, such as alpha-fetoprotein (AFP) or protein induced by vitamin K absence or antagonists-II (PIVKA-II), and ultrasonography. However, AFP and PIVKA-II are limited in their ability to indicate early-stage HCC.

Alterations in the oligosaccharide structure of serum proteins have been associated with the development of HCC. For example, α-1,6 fucosylation (core fucosylation) of AFP (AFP-L3) is increased in and serves as a clinical marker of HCC [3,4,5]. However, due to its sensitivity of approximately 65%, AFP-L3 is an insufficient marker for detection of early-stage cancer [6]. Hence, other studies have searched for alternative markers by focusing on other serum glycoproteins [7,8,9,10]. Glycoprotein isolation and *N*-glycan characterization require multiple steps of protein purification and sugar chain structure determination. Structural alterations of protein glycans in whole serum are often observed in cancer patients and thus are also putative biomarker candidates. Assessment of these alterations requires no protein purification and is comparatively easy to perform, although detection of altered oligosaccharides in sera remains challenging. The efficacy of immunological or lectin (proteins showing binding activity to sugar chains) affinity screens is limited, because antibodies and lectins recognize only partial oligosaccharide structures.

A comprehensive and quantitative method to analyze *N*-linked oligosaccharides using two-dimensional mapping was described previously [11,12]. However, the complex sample preparation and extended analytical period render this method inappropriate for analyzing large numbers of samples. Typically, sialic acid residues are removed from the *N*-glycans prior to analysis, because they lower the ionization efficacy of the mass spectrometry (MS) and complicate high-performance liquid chromatography (HPLC). Nevertheless, as most serum *N*-glycans are sialylated, it is crucial to retain information about their sialylation status when searching for *N*-glycan markers.

To resolve these issues, we developed a new technique to easily estimate the sialylated portion of a sugar chain, which allows quantification of sialylated and non-sialylated *N*-glycans in whole serum [13,14,15]. This optimization enabled us to obtain the absolute (rather than relative) amount of a single type of sugar chain in serum. In this study, we characterized the absolute quantities of serum sialylated and non-sialylated *N*-glycans and identified several novel *N*-linked oligosaccharide markers that can distinguish patients with early-stage HCC from cirrhotic patients. Among the markers, one *N*-glycan, named A2G1(6)FB, was detected in HCV-infected patients with early-stage HCC. We further demonstrated that A2G1(6)FB is harbored on immunoglobulin (Ig). Together, our results suggest that A2G1(6)FB may be a potential marker for patients with HCC at early stages.

## 2. Results

### 2.1. Experimental Design

**Figure 1:**
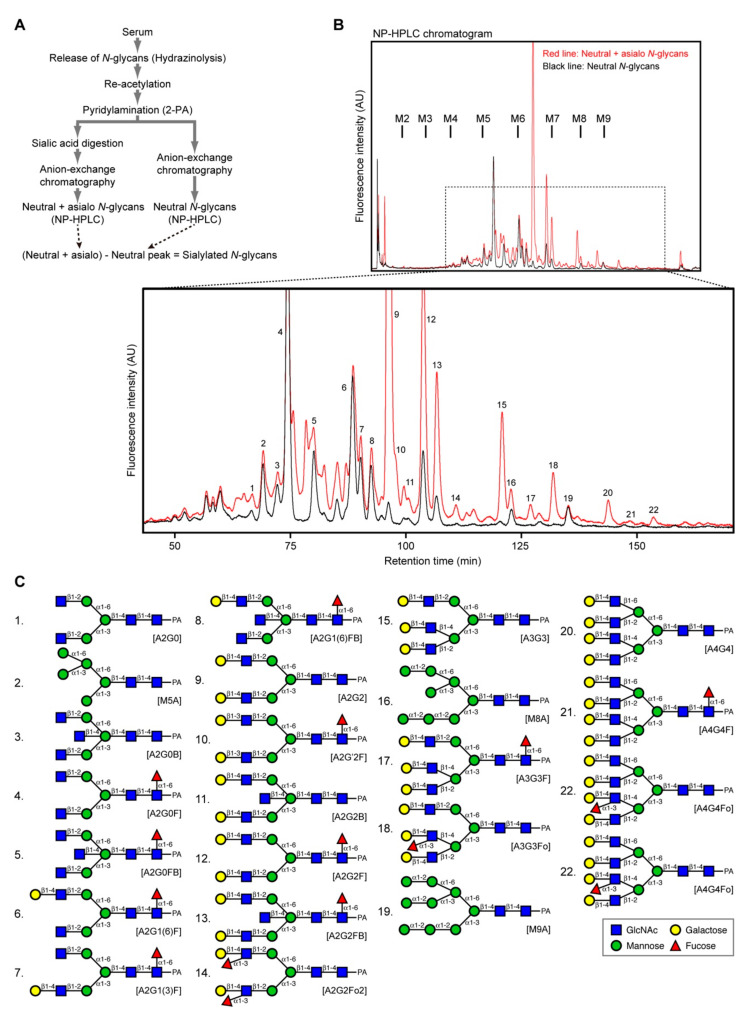
Scheme for human serum *N*-glycan preparation and profiling. (**A**) Serum *N*-glycan analysis strategy. (**B**) Chromatogram of normal-phase (NP)-HPLC shows the fluorescence-labeled neutral + asialo *N*-glycans (red line) and neutral *N*-glycans (black line). Elution positions of external standards (mannose-units: M2–M9) are shown. The lower panel shows the enlarged chromatogram between retention times 40 and 180 min. (**C**) The *N*-glycans of peaks 1–22 in Figure 1B were identified. All structures are shown as pyridylaminated (PA) forms.

To identify the *N*-glycans in serum, these were released from proteins by hydrazinolysis and fluorescently labeled by pyridylamination (Figure 1A). Half of the sample was treated with neuraminidase to remove sialic acid residues. The untreated half was applied to a DE52 anion exchange column, and the non-adsorbed fraction (the neutral sugar chain fraction) was collected. The neuraminidase-treated sample was similarly processed, and the non-adsorbed fraction (the neutral + asialo sugar chain fraction) was obtained. Both fractions were subjected to normal-phase (NP) HPLC. The difference between the neutral + asialo sugar chains (red line) and neutral sugar chains (black line) represents the original proportion of sialylated sugar chains. In total, we detected 54 chromatograph peaks from a whole-serum sample (Figure 1B). Twenty-two *N*-glycans were identified by 2D mapping consisting of NP-HPLC and reverse-phase (RP) HPLC or exoglycosidase digestion (Figure 1C).

### 2.2. *N*-Glycans Remain Stable in Sera for at Least One Day

As sialic acid residues can be easily removed from sugar chains, we first examined the stability of sialylated sugar chains in sera. Plasma *N*-glycan profiles are quite stable within an individual when drawing blood over the course of five days [16]. However, the stability of *N*-glycans in serum was unknown. Freshly prepared human sera were kept at room temperature for 0.5, 3, 6, 9, or 24 h, and *N*-glycans were prepared and analyzed by MonoQ anion-exchange chromatography to determine the stability of sialylated *N*-glycans (Figure S1A, Supplementary Materials). The amount of neutral (S0), mono- (S1), di- (S2), and tri- (S3) sialylated *N*-glycans remained unchanged over 24 h (Figure S1B). Desialylated *N*-glycans were analyzed by NP-HPLC after neuraminidase treatment to assess the oligosaccharide structure, and no obvious changes were detected (Figure S1C). Thus, sialylated *N*-glycans are stable in sera for at least one day. In addition, there were no effects of freezing–thawing on the stability of sialylated *N*-glycans nor of the daily variation of sugar chain expression levels, in particular of food intake, on sialylated *N*-glycan profiling (data not shown).

### 2.3. *N*-Glycan Recovery Rate Is Highly Reproducible

To test the reproducibility of our system, we selected three *N*-glycans showing peaks that were relatively isolated and therefore more easily quantified. Evaluation of the reproducibility of the recovered *N*-glycans was repeated six times by focusing on three major *N*-glycan peaks. The *N*-glycan recovery rate was demonstrated to be reproducible (Table S1), with a 6% coefficient of variance. Therefore, we used the absolute value of each peak throughout our analysis.

### 2.4. *N*-Glycan Profiles in Human Sera

To identify *N*-glycans that are increased in patients with HCC but not in cirrhotic patients, we examined non-sialylated and sialylated *N*-glycan profiles in sera from healthy controls (*n* = 71) and HCV-infected patients with hepatitis (*n* = 11), LC (*n* = 24), HCC (*n* = 76), gastric cancer (*n* = 12), pancreatic cancer (*n* = 12), other gastrointestinal cancers (*n* = 15), and other gastrointestinal diseases (*n* = 25) (Table 1). Distinct changes in neutral *N*-glycans were identified in sera from patients with LC and HCC compared with healthy controls, including A2G0, M5A, and biantennary *N*-glycans containing bisecting GlcNAc residues (A2G0B, A2G1(6)FB, and A2G2B) (Figure S2). Among the sialylated *N*-glycans, biantennary sugar chains containing bisecting GlcNAc (A2G2B) or outer fucose (A2G2Fo2) were also higher in patients with HCC than in healthy controls (Figure S2).

### 2.5. Exploring *N*-Glycans as Potential Early-Stage HCC Markers

After obtaining NP-HPLC chromatograms, we quantified peak areas, normalized them relative to the amount of starting acetone precipitate, and calculated the absolute amount of *N*-glycans in acetone-precipitated human serum samples. The levels of five *N*-glycans (A2G0, M5A, A2G0B, A2G1(6)FB, and A2G2B, all *p* < 0.0001, unpaired *t*-test) and one sialylated *N*-glycan (A2G2Fo2, *p* < 0.0001, unpaired *t*-test) were significantly higher in sera from HCV-infected patients with HCC than control sera (Table 1).

Moreover, significantly greater levels of several *N*-glycans were found in sera from HCV-infected patients with HCC than in those with LC or in patients with hepatitis (A2G0, *p* = 0.0086; M5A, *p* < 0.0001; A2G0B, *p* = 0.0004; A2G1(6)FB, *p* < 0.0001; A2G2B, *p* = 0.0001; and A2G2Fo2, *p* = 0.0039; unpaired *t*-test) (Table 1).

### 2.6. A2G1(6)FB N-Glycan Marks Early-Stage HCC

**Figure 2:**
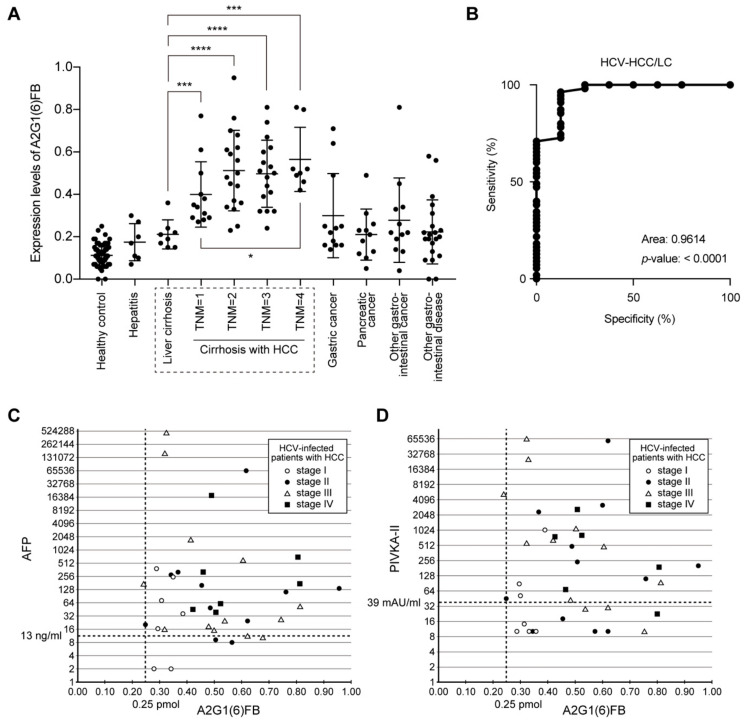
Serum A2G1(6)FB is a potential candidate for primary hepatocellular carcinoma (HCC) in hepatitis C virus (HCV)-dependent cirrhosis. (**A**) Scatter plots showing A2G1(6)FB level in human sera. A2G1(6)FB expression levels were significantly higher in the HCC group compared with the liver cirrhosis (LC) group. * *p* < 0.05, *** *p* < 0.001, **** *p* < 0.0001, Mann–Whitney test. Dotted line indicates the LC patients with or without HCC. (**B**) Receiver operating characteristic (ROC) curves for A2G1(6)FB. The area under the curve (AUC) of A2G1(6)FB was 0.9614 (*p* < 0.0001) in HCV-infected patients with HCC compared with LC (see also Tables S2 and S3). (**C**,**D**) Correlation between A2G1(6)FB and either AFP or PIVKA-II. The thresholds were defined as 13 ng/mL for AFP (**C**) and 39 mAU/mL for PIVKA-II (**D**) in patients with HCC. There was no correlation between levels of A2G1(6)FB and AFP or PIVKA-II.

Next, we addressed whether the identified *N*-glycans were associated with early-stage HCC. HCV-infected patients with HCC were classified as stage I (*n* = 12), stage II (*n* = 18), stage III (*n* = 17), or stage IV (*n* = 8). A2G1(6)FB expression levels were significantly higher in patients at all stages (stage I, *p* < 0.001; stage II, *p* < 0.0001; stage III, *p* < 0.0001; and stage IV, *p* < 0.001; Mann–Whitney test) than in cirrhotic patients without HCC (Figure 2A). Therefore, neutral *N*-glycan A2G1(6)FB may be a potential marker for the detection of early-stage HCC in HCV-infected patients with LC.

We analyzed the correlation between age and A2G1(6)FB expression in HCV-infected HCC patients (Figure S3). There was no significant correlation between age and A2G1(6)FB expression levels. A2G1(6)FB expression levels were also examined in the groups classified using the Child–Pugh score (Figure S4).

### 2.7. Sensitivity and Specificity of A2G1(6)FB in HCC

The receiver operating characteristic (ROC) curve was plotted to determine the sensitivity and specificity of A2G1(6)FB in differentiating HCC from cirrhosis without HCC among HCV-infected patients (Figure 2B). The area under the curve (AUC) for A2G1(6)FB was 0.9614 (*p* < 0.0001) in HCV-infected patients with HCC compared with those with LC (Tables S2 and S3). Thus, A2G1(6)FB has a better accuracy than currently used markers, such as AFP with a diagnostic accuracy of 60%, demonstrating that it may be a promising marker for patients with early-stage HCC.

### 2.8. No Correlation between A2G1(6)FB and AFP or PIVKA-II

We further defined the optimal cut-off value of A2G1(6)FB from ROC curve analysis, and compared its diagnostic efficacy with that of currently used markers by plotting its levels against those of AFP or PIVKA-II (Figure 2C,D). In our study group, 6 out of 38 HCC patients were AFP-negative (<13 ng/mL) and 12 out of 37 were PIVKA-II-negative (<39 mAU/mL) (cut-off values were chosen according to clinical criteria used at Kagawa University Hospital). In contrast, only two HCC patients were A2G1(6)FB-negative. Interestingly, there was no apparent correlation between A2G1(6)FB and AFP (stage I: r = −0.09694, *p* = 0.84; stage II: r = 0.1236, *p* = 0.72; stage III: r = −0.4076, *p* = 0.17; stage IV: r = −0.1996, *p* = 0.67, Pearson’s r) or PIVKA-II (stage I: r = 0.6777, *p* = 0.09; stage II: r = 0.1118, *p* = 0.73; stage III: r = −0.4462, *p* = 0.15; stage IV: r = −0.4176, *p* = 0.41, Pearson’s r) (Figure 2C,D and Figure S5). Therefore, simultaneous use of A2G1(6)FB levels and traditional marker levels may provide a more accurate diagnosis of HCC than individual markers alone.

### 2.9. A2G1(6)FB Is Harbored on Ig Heavy Chain

**Figure 3:**
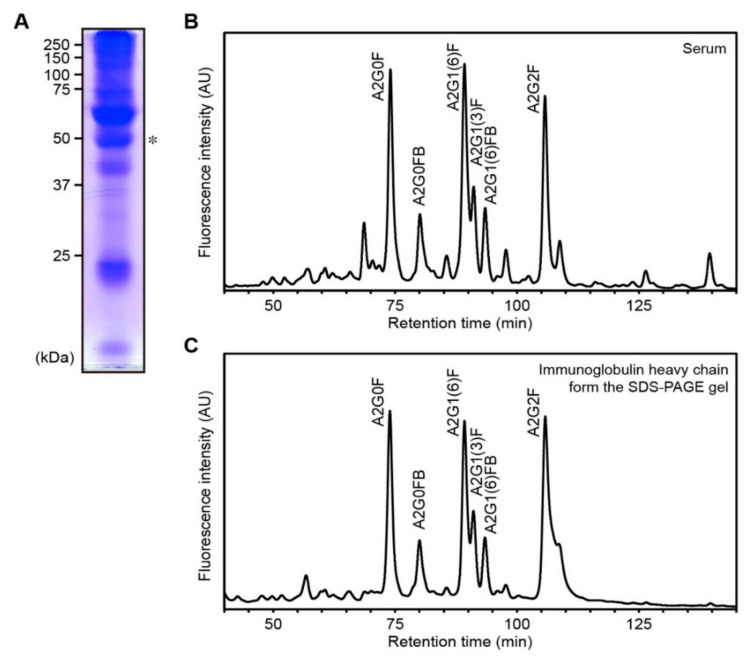
A2G1(6)FB is harbored on the Ig heavy chain. (**A**) Coomassie Brilliant Blue (CBB) staining of acetone-precipitated serum sample (healthy control). * The band (asterisk) was excised and subjected to *N*-glycan analysis. (**B**) NP-HPLC chromatogram of neutral *N*-glycans in healthy control serum. (**C**) NP-HPLC chromatogram of neutral *N*-glycans from the gel piece in Figure 3A. The band mainly contained Ig heavy chain.

Finally, we separated acetone-precipitated serum samples by sodium dodecyl sulfate polyacrylamide gel electrophoresis (SDS-PAGE) to identify serum proteins harboring A2G1(6)FB. After Coomassie Brilliant Blue (CBB) staining, the Ig heavy-chain band (around 50 kDa) was excised from the SDS-PAGE gel because A2G1(6)FB was previously detected from purified human Ig heavy chain using our method [15] (Figure 3A). Sera from four patients with low to high levels of A2G1(6)FB were analyzed. The profile of the major neutral *N*-glycans, including A2G1(6)FB from the gel, mainly containing Ig heavy chain, was similar to that from serum (Figure 3B,C). These results suggest that A2G1(6)FB is harbored on the Ig heavy chain, and the serum levels of A2G1(6)FB are mainly derived from the Ig heavy chain.

## 3. Discussion

Here, we identified several *N*-glycans that are significantly increased in HCV-infected HCC patients. A2G1(6)FB was identified as a potential marker for HCC for the following reasons: (1) the peak on a chromatogram was isolated from other peaks, making its quantification reliable and simple, (2) AUC of 96.14%, and (3) no correlation with AFP or PIVKA-II.

### 3.1. Comparison with Other *N*-Glycan Markers for HCC

Miura et al. [17] reported that the A2G1(3 and 6)B/M7, M5/M7, and A2G1(3 and 6)B/A2G0 ratios can be used to diagnose HCC. Consistent with these findings, we observed that M5A levels were increased in HCV-infected patients with HCC. M5A tended to increase from an early stage of HCC development (Table 1; LC with HCV: 0.216 ± 0.113; stage I HCC with HCV: 0.457 ± 0.115). The M5A peak was also isolated from other peaks, making its quantification reliable and simple. Therefore, M5A may be another potential marker for HCV-infected patients with HCC.

Recently, the same authors reported glycomic analysis using matrix-assisted laser desorption/ionization time-of-flight (MALDI-TOF) MS as a tool to identify the *N*-glycans released from serum glycoproteins by PNGase F treatment for novel biomarkers of HCC [18]. The levels of 14 *N*-glycans were higher in the sera of patients with HCC compared with controls; however, only M5A was increased in sera from HCC patients in both studies [17,18].

The A2G1(6)FB oligosaccharide harbors core fucose and bisecting GlcNAC residues and thus can be recognized by *Lens culinaris* agglutinin (LCA, for core fucose detection) and erythroagglutinating phytohemagglutinin (E4-PHA, for bisecting GlcNAc detection). LCA-reactive AFP-L3 and E4-PHA-reactive AFP are frequently increased in HCC but not in hepatitis or cirrhosis, and can be observed before tumors are detectable by imaging [19]. Moreover, previous studies have shown higher serum *N*-acetylglucosaminyltransferase III (Gnt-III) activities in patients with HCC compared with those in patients with chronic hepatitis or healthy controls [20,21]. However, the sensitivity and specificity of these markers were not as high as those of A2G1(6)FB.

### 3.2. Characterization and structure of *N*-glycan A2G1(6)FB

Immunoglobulin G (IgG) has one *N*-glycan attachment site on each of its two heavy chains. The function of IgG has been shown to vary depending on its *N*-glycan structure [22] and is often used as a prognostic marker for cancer [23]. Galactosylation, sialylation, bisecting GlcNAc addition, and core fucosylation of *N*-glycans affect IgG function and cancer prognosis.

In this study, we found that A2G1(6)FB was carried by the Ig heavy chain. Interestingly, Ig has one galactose, bisecting GlcNAc, and core fucose, but no sialylic acid. The value of peak areas in HPLC chromatograms represents the amount of A2G1(6)FB per mg protein (not IgG). Thus, this value is not only influenced by the change in the *N*-glycan composition of IgG, but also by the amount of IgG within the serum proteins. Moreover, there was no apparent correlation between A2G1(6)FB and AFP or PIVKA-II. The average ages of healthy control patients (44.1 years old) and those with LC (65.9 years old) or liver cancer (72.3 years old) were statistically different (one-way ANOVA) (Table 1). As there were statistical age differences between the groups, we found that there was no significant correlation between age and A2G1(6)FB expression (Figure S3). The percentage of A2G1(6)FB in total IgG glycans increases with age; however, its average increase was only from 5% to 6% in a Han Chinese population between 40 and 70 years of age [24]. Therefore, the difference in the average age between the three groups should exert negligible effects on the basal level of A2G1(6)FB.

Identification of genetic loci in human IgG associated with harboring *N*-glycans revealed that A2G1(6)FB is associated with MGAT3 (GnT-III) and SMARCB1-DERL3 [25]. As A2G1(6)FB has a bisecting GlcNAc residue, it is reasonable to associate it with MGAT3, the enzyme responsible for conjugating bisecting GlcNAc. DERL3 is involved in the endoplasmic reticulum-associated degradation machinery and functions as a tumor suppressor [26]. DERL3 is also downregulated in several primary tumors, including HCC [26], suggesting that alteration in DERL3 activity is connected with altered serum A2G1(6)FB levels and HCC induction. Although SMARCB1 mutations are also related to various tumors, no studies have demonstrated its involvement in HCC. Further understanding of the role of A2G1(6)FB should give rise to novel insights and therapeutic targets in HCC.

The present study had some limitations. The sample size was low. Therefore, further studies using larger validation cohorts of patients are required in the future. Furthermore, although A2G1(6)FB expression levels are high in liver cancer, the relationship between A2G1(6)FB expression and liver function remains unclear and should be addressed in future studies (Figure S4).

In conclusion, the *N*-glycan A2G1(6)FB may be a potential biomarker to detect early HCC development in HCV-infected patients.

## 4. Materials and Methods

### 4.1. Patients, Ethics, and Clinical Diagnosis

This study was approved in advance by the Ethics Committees of the National Institute for Physiological Sciences, Kagawa University, Kita-gun, Japan, and Mitsubishi Chemical Group Science and Technology Research Center, Yokohama, Japan (approval numbers: 11B004, 6 May 2011; 12B008, 21 May 2012; Heisei16-15, 1 December 2009; Heisei22-86, 21 February 2011; 100113-2, 20 January 2010). All subjects provided written informed consent prior to participation. All patients were enrolled at Kagawa University Hospital (Kita-gun, Kagawa, Japan). Data regarding HCV-related patients with HCC who had chronic hepatitis and cirrhosis were collected, and the patients with HCV-related chronic hepatitis and cirrhosis without HCC were selected based on image diagnosis and histopathology. A pathological examination of liver biopsies was performed to confirm LC. HCC was diagnosed based on the findings from dynamic CT, gadolinium-ethoxybenzyl-diethylenetriamine pentaacetic acid-enhanced magnetic resonance imaging (EOS-MRI), and liver biopsy testing. Typical HCC pattern imaging criteria were defined as follows: (i) hypervascularity was defined as focal lesion hyperattenuation relative to the liver during the arterial phase, and wash-out appearance was observed during the portal and parenchymal phase; and (ii) tumors were revealed as defects in the hepatobiliary phase of EOB-MRI. HCV infection was defined as detectable HCV RNA in serum. Serum HCV RNA levels were measured using a real-time polymerase chain reaction-based method (lower limit of detection, 1.2 log_10_ IU/mL; COBAS TaqMan HCV Test 2.0, Roche Diagnostics, Rotkreuz, Switzerland). Sera were obtained from patients who were diagnosed with liver disease before therapy. The sera obtained from patients were immediately stored in a −80 °C freezer and transported to our facility at −20 °C for use in the present study. Tumor stage was ranked according to the tumor–node–metastasis (TNM) criteria as follows: stage I = single lesion < 2 cm in diameter; stage II = solitary with vascular invasion, multiple < 5 cm; stage III = multiple > 5 cm, invading the major branch of portal or hepatic veins; and stage IV, invading adjacent organs other than the gallbladder, perforating visceral peritoneum.

### 4.2. Sample Preparations

Human sera from subjects with no obvious liver diseases were obtained from Soiken Holdings Inc., Tokyo, Japan. Cold acetone (9 mL) was added to 1 mL of serum; the sample was mixed well and centrifuged at 4000× *g* for 20 min at 4 °C. The resulting pellet was dried in a vacuum evaporator (DC400, Yamato Scientific Co., Ltd., Tokyo, Japan) and stored at −20 °C until use. Pyridylaminated (PA) *N*-glycans [13,14,15] and neutral and desialylated *N*-glycans [13,14,15,27,28,29,30,31,32] were prepared as previously described. Briefly, for PA-*N*-glycans, a lyophilized sample (2 mg) was heated with anhydrous hydrazine to release sugar chains, eluted using a graphite carbon column, and dried to obtain the *N*-acetylated oligosaccharides. For neutral and desialylated *N*-glycans, purified glycans were tagged with the fluorophore, 2-aminopyridine. The PA-oligosaccharides were divided into two equal volumes. One half was eluted with a DE52 column and collected, and the other was treated with neuraminidase and applied to a DE52 column.

### 4.3. *N*-Glycan Analyses

HPLC analyses of PA-*N*-glycans were performed as described previously [13,15,32]. SDS-PAGE with CBB staining and *N*-glycan analysis from SDS-PA gels were also performed based on published methods [15]. Briefly, fluorescent-tagged glycans were analyzed by NP-HPLC with an NP column or RP-HPLC with an RP column. Glycan structures were identified by calculating the mannose unit value in NP-HPLC and the glucose unit value in RP-HPLC as previously described [28,30] or by comparison with known standards and sequential exoglycosidase digestion.

### 4.4. Data Quantification and Statistical Analysis

NP- and RP-HPLC chromatogram data were analyzed by LC station software (Shimadzu, Kyoto, Japan) and Empower2 software (Waters, Milford, MA, USA), respectively. Statistical analyses (unpaired *t*-test, Mann–Whitney test, and ROC curve) were performed using GraphPad Prism 7 (GraphPad Software, San Diego, CA, USA). *p* < 0.05 was considered to indicate statistical significance.

## Figures and Tables

**Table 1:** Patient characteristics and serum expression levels of six *N*-glycans.

		Healthy Control		Hepatitis		Liver Cirrhosis	Liver Cancer	Gastric Cancer	Pancreatic Cancer	Other Gastrointestinal Cancers	Other Gastrointestinal Diseases
**Number**		71		11		24	76	12	12	15	25
**Etiology** **(HBV/HCV/other)**				0/7/4		3/8/13	9/55/12				
**Tumor stage** **(HBV/HCV/other)**	**T1**						2/12/3				
	**T2**						3/18/4				
	**T3**						1/17/2				
	**T4**						3/8/3				
**Age (years)**		44.1 ± 13.1		53.6 ± 9.9		65.9 ± 9.4	72.3 ± 6.5	70.8 ± 7.6	64.8 ± 11.3	67.2 ± 13.3	71.1 ± 10.1
**AST (IU/L)**		20.2 ± 6.3		144.0 ± 484.7		59.5 ± 40.5	76.9 ± 69.6	20.0 ± 5.2	93.9 ± 140.3	48.9 ± 51.6	32.4 ± 29.0
**ALT (IU/L)**		18.5 ± 14.2		103.3 ± 248.9		36.4 ± 21.5	55.5 ± 63.1	19.9 ± 8.0	182.6 ± 272.5	58.9 ± 101.7	30.6 ± 46.5
**Albumin (g/dL)**		4.5 ± 0.2		4.2 ± 0.5		3.2 ± 0.6	3.1 ± 0.6	4.1 ± 0.3	3.6 ± 0.3	3.5 ± 0.1	3.7 ± 0.7
**Total serum** **protein (g/dL)**		7.1 ± 0.3		7.1 ± 0.5		7.2 ± 0.6	6.9 ± 0.8	6.7 ± 0.6	6.5 ± 0.7	6.6 ± 0.8	6.4 ± 0.5
**Total bilirubin (mg/dL)**		1.0 ± 0.4		0.9 ± 0.3		2.3 ± 2.2	1.7 ± 1.8	0.5 ± 0.2	3.8 ± 4.6	2.4 ± 4.0	1.5 ± 2.7
**AFP (ng/mL)**				11.5 ± 8.1		12.0 ± 16.0	18,202.7 ± 73,224.8				
**PIVKA-II** **(mAU/mL)**				30.7 ± 23.3		172.9 ± 417.3	3692.4 ± 12,058.9				
**CEA (ng/mL)**								3.2 ± 1.2	4.2 ± 3.2	23.2 ± 59.0	
**CA19-9 (U/mL)**								165.9 ± 441.3	5987.2 ± 14,636.8	18,955.0 ± 55,958.0	
***N*-glycan** **(type)**	**Healthy** **control**				**Hepatitis**	**Liver** **cirrhosis**	**Liver** **cancer**	**Gastric** **cancer**	**Pancreatic** **cancer**	**Other** **gastrointestinal** **cancers**	**Other** **gastrointestinal** **diseases**
**A2G0** **(neutral)**	0.033 ± 0.017		**HBV**		n.d.	0.227 ± 0.148	0.180 ± 0.100	0.096 ± 0.070	0.103 ± 0.061	0.115 ± 0.084	0.119 ± 0.104
			**HCV**		0.065 ± 0.037	0.090 ± 0.068	0.214 ± 0.126				
			**other**		0.077 ± 0.014	0.220 ± 0.118	0.209 ± 0.136				
**M5A** **(neutral)**	0.193± 0.063		**HBV**		n.d.	0.512 ± 0.148	0.541 ± 0.201	0.360 ± 0.104	0.324 ± 0.130	0.416 ± 0.199	0.273 ± 0.145
			**HCV**		0.265 ± 0.090	0.216 ± 0.113	0.507 ± 0.128				
			**other**		0.313 ± 0.066	0.597 ± 0.264	0.413 ± 0.106				
**A2G0B** **(neutral)**	0.060 ± 0.022		**HBV**		n.d.	0.565 ± 0.326	0.363 ± 0.196	0.157 ± 0.077	0.180 ± 0.102	0.224 ± 0.156	0.149 ± 0.137
			**HCV**		0.129 ± 0.034	0.131 ± 0.114	0.374 ± 0.176				
			**other**		0.147 ± 0.027	0.739 ± 0.342	0.351 ± 0.207				
**A2G1(6)FB** **(neutral)**	0.112 ± 0.047		**HBV**		n.d.	0.562 ± 0.288	0.459 ± 0.256	0.299 ± 0.200	0.211 ± 0.121	0.275 ± 0.186	0.223 ± 0.151
			**HCV**		0.175 ± 0.087	0.210 ± 0.068	0.489 ± 0.176				
			**other**		0.236 ± 0.093	0.538 ± 0.238	0.521 ± 0.208				
**A2G2B** **(neutral)**	0.025 ± 0.011		**HBV**		n.d.	0.049 ± 0.009	0.057 ± 0.016	0.053 ± 0.025	0.034 ± 0.017	0.046 ± 0.027	0.033 ± 0.018
			**HCV**		0.031 ± 0.018	0.024 ± 0.012	0.058 ± 0.023				
			**other**		0.037± 0.002	0.054± 0.010	0.043± 0.012				
**A2G2Fo2** **(neutral+asialo)**	0.056 ± 0.031		**HBV**		n.d.	0.254 ± 0.109	0.251 ± 0.182	0.134 ± 0.082	0.228 ± 0.142	0.206 ± 0.265	0.230 ± 0.082
			**HCV**		0.169 ± 0.204	0.084 ± 0.039	0.231 ± 0.137				
			**other**		0.370 ± 0.082	0.281 ± 0.115	0.209 ± 0.147				

T1, tumor–node–metastasis (TNM) criteria stage I; T2, TNM criteria stage II; T3, TNM criteria stage III; T4, TNM criteria stage IV; HBV, hepatitis B virus; HCV, hepatitis C virus; other, non-HBV and non-HCV antigen; AST, aspartate aminotransferase; ALT, alanine aminotransferase; AFP, alpha-fetoprotein; PIVKA-II, protein induced by vitamin K absence or antagonists-II; CEA, carcinoembryonic antigen; CA19-9, carbohydrate antigen 19-9; n.d., no data.

## Supplementary Materials

The following are available online at https://www.mdpi.com/1422-0067/21/23/8913/s1.

## Abbreviations

AFP	alpha-fetoprotein
AU	arbitrary unit
AUC	area under the curve
CBB	Coomassie Brilliant Blue
E4-PHA	erythroagglutinating phytohemagglutinin
GnT	*N*-acetylglucosaminyltransferase
HBV	hepatitis B virus
HCC	hepatocellular carcinoma
HCV	hepatitis C virus
HPLC	high-performance liquid chromatography
IgG	immunoglobulin G
LC	liver cirrhosis
LCA	*Lens culinaris* agglutinin
NP	normal-phase
PA	pyridylaminated
PIVKA-II	protein induced by vitamin K absence or antagonists-II
ROC	receiver operating characteristic
RP	reverse-phase
SDS-PAGE	sodium dodecyl sulfate polyacrylamide gel electrophoresis
