# Supplementary material for: A Potential Serum N-glycan Biomarker for Hepatitis C Virus-Related Early-Stage Hepatocellular Carcinoma with Liver Cirrhosis
Source: Int J Mol Sci. 2020 Nov 24;21(23):8913. doi: 10.3390/ijms21238913 (PMC7727814; doi:10.3390/ijms21238913)
Supplement: Supplementary file 1 [file ijms-21-08913-s001.pdf]

International Journal of *Molecular Sciences*

MDPI

Supplementary Materials

A Potential Serum *N*-glycan Biomarker for Hepatitis C Virus-Related Early-Stage Hepatocellular Carcinoma with Liver Cirrhosis

Mikito Higashi [1,2,†], Takeshi Yoshimura [1,3,4,†,*], Noriyoshi Usui [5,6], Yuichiro Kano [1], Akihiro Deguchi [7], Kazuhiro Tanabe [2], Youichi Uchimura [2,¶], Shigeki Kuriyama [7,¶], Yasuyuki Suzuki [8], Tsutomu Masaki [7] and Kazuhiro Ikenaka [1,3,¶,*]

1 Division of Neurobiology and Bioinformatics, National Institute for Physiological Sciences, National Institutes of Natural Sciences, Okazaki, Aichi 444-8787, Japan
2 Mitsubishi Chemical Group Science and Technology Research Center, Yokohama, Kanagawa 227-8502, Japan
3 Department of Physiological Sciences, School of Life Sciences, SOKENDAI (The Graduate University for Advanced Studies), Hayama, Kanagawa 240-0193, Japan
4 Department of Child Development and Molecular Brain Science, United Graduate School of Child Development, Osaka University, Suita, Osaka 565-0871, Japan
5 Department of Neuroscience and Cell Biology, Graduate School of Medicine, Osaka University, Suita, Osaka 565-0871, Japan
6 Addiction Research Unit, Osaka Psychiatric Research Center, Osaka Psychiatric Medical Center, Osaka 541-8567, Japan
7 Department of Gastroenterology and Neurology, Faculty of Medicine, Kagawa University, Kita-gun, Kagawa 761-0793, Japan
8 Department of Gastroenterological Surgery, Faculty of Medicine, Kagawa University, Kita-gun, Kagawa 761-0793, Japan
* Correspondence: tyoshimu@ugscd.osaka-u.ac.jp (T.Y.); ikenaka@nips.ac.jp (K.I.)
† These authors contributed equally to this work.
¶ Deceased.

Supplementary Materials and Methods

Sample preparations

Human sera from subjects with no obvious liver diseases were obtained from Soiken Holdings Inc, Tokyo, Japan. Cold acetone (9 mL) was added to 1 mL of serum; the sample was mixed well and centrifuged at 4,000 × g for 20 min at 4 °C. Preparations of pyridylaminated *N*-glycans [1-3] and neutral and desialylated *N*-glycans [1-9] were performed as previously described. For pyridylaminated *N*-glycans, a lyophilized sample (2 mg) was heated with 200 µL anhydrous hydrazine (Tokyo Chemical Industry, Tokyo, Japan) at 100 °C for 10 h to release sugar chains. The hydrazine solution was then mixed with 3 mL 50 mM ammonium acetate buffer (pH 7.0) and loaded onto a graphite carbon column (GL Science, Tokyo, Japan). Oligosaccharides were eluted with 5 mL of a mixed solution (20 mM triethylamine acetate buffer (pH 7.0) and 60% acetonitrile containing 2% acetic anhydride). The eluted solution was dried using a SpeedVac to obtain the *N*-acetylated oligosaccharides. For neutral and desialylated *N*-glycans, purified glycans were tagged with a fluorophore, 2-aminopyridine. Excess reagent was removed with a cellulose cartridge column (Takara Bio, Ohtsu, Japan). Pyridylaminated (PA)-oligosaccharides were divided into two equal volumes. One half was applied to a DE52 column (Whatman, GE Healthcare, Little Chalfont, UK) equilibrated with water adjusted to pH 9.0 by 1 M aqueous ammonia [1]; the non-adsorbed fraction was collected. The other half was treated with neuraminidase (Nacalai Tesque, Kyoto, Japan) and applied to a DE52 column.

N-glycan analyses

Analyses of PA-*N*-glycans using HPLC were performed as described previously [1,3,9]. Sodium dodecyl sulfate-polyacrylamide gel electrophoresis (SDS-PAGE) with Coomassie Brilliant Blue (CBB) staining and *N*-glycan analysis from SDS-PAGE gels were also performed as described previously [3]. Fluorescent-tagged glycans were analyzed by HPLC using a NP column (Shodex Asahipak NH2P-50 4E, 4.6 × 250 mm, Showa Denko, Tokyo, Japan). The mobile phase consisted of solvent A (50 mM ammonium acetate buffer containing 93% acetonitrile, pH 7.0) and solvent B (50 mM ammonium acetate buffer containing 20% acetonitrile, pH 7.0) and the following gradient: linear gradient from 25–42% solvent B at 0.6 mL/min for 180 min followed by a linear gradient from 52–100% solvent B for the next 3 min. NP-HPLC analysis was performed using the Prominence HPLC system with a fluorescence detector (excitation and emission wavelengths of 310 nm and 380 nm, respectively) (Shimadzu, Kyoto, Japan). Each detected PA-*N*-glycan was further analyzed by RP-HPLC using a Develosil C30-UG-5 column (4.6 × 150 mm, Nomura Chemical, Seto, Japan). The mobile phase consisted of solvent A (5 mM ammonium acetate, pH 4.0) and solvent B (10% acetonitrile with solvent A). The flow rate was 0.5 mL/min and monitored by a fluorescence detector (FP-2025 Plus, JASCO, Hachioji, Japan) using excitation and emission wavelengths of 320 nm and 400 nm, respectively. Glycan structures were identified by calculating the Mannose-Unit value in NP-HPLC and the Glucose-Unit value in RP-HPLC as previously described [5,7] or by comparison to known standards and sequential exoglycosidase digestion. Anion-exchange chromatography was performed using a MonoQ HR5/5 column (5 × 50 mm, GE Healthcare) at a flow rate of 1.0 mL/min at room temperature. Solvent C was distilled H_2O titrated to pH 9.0 with 1 M aqueous ammonia, and solvent D was 0.5 M ammonium acetate (pH 9.0). The column was equilibrated with solvent C. After injecting a sample, the proportion of solvent D was increased linearly to 12% in 3 min, to 40% in 14 min, and to 100% in 5 min. PA-sugar chains were detected at an excitation wavelength of 310 nm and emission wavelength of 380 nm (FP-2025 Plus, JASCO).

Data quantification and statistical analysis

NP-HPLC and RP-HPLC chromatogram data were analyzed with LC station software (Shimadzu) and Empower2 software (Waters, Milford, MA, USA), respectively. Statistical analyses (ROC curve and Pearson's r) were performed using GraphPad Prism7.

Supplementary References

1. Torii, T.; Yoshimura, T.; Narumi, M.; Hitoshi, S.; Takaki, Y.; Tsuji, S.; Ikenaka, K. Determination of major sialylated N-glycans and identification of branched sialylated N-glycans that dynamically change their content during development in the mouse cerebral cortex. *Glycoconj. J.* **2014**, *31*, 671-683.
2. Tanabe, K.; Ikenaka, K. In-column removal of hydrazine and N-acetylation of oligosaccharides released by hydrazionolysis. *Anal. Biochem.* **2006**, *348*, 324-326.
3. Yoshimura, T.; Yamada, G.; Narumi, M.; Koike, T.; Ishii, A.; Sela, I.; Mitrani-Rosenbaum, S.; Ikenaka, K. Detection of N-glycans on small amounts of glycoproteins in tissue samples and sodium dodecyl sulfate-polyacrylamide gels. *Anal. Biochem.* **2012**, *423*, 253-260.
4. Hase, S.; Natsuka, S.; Oku, H.; Ikenaka, T. Identification method for twelve oligomannose-type sugar chains thought to be processing intermediates of glycoproteins. *Anal. Biochem.* **1987**, *167*, 321-326.
5. Hase, S. High-performance liquid chromatography of pyridylaminated saccharides. *Methods Enzymol.* **1994**, *230*, 225-237.
6. Fujimoto, I.; Menon, K.K.; Otake, Y.; Tanaka, F.; Wada, H.; Takahashi, H.; Tsuji, S.; Natsuka, S.; Nakakita, S.i.; Hase, S.; Ikenaka, K. Systematic analysis of N-linked sugar chains from whole tissue employing partial automation. *Anal. Biochem.* **1999**, *267*, 336-343.
7. Otake, Y.; Fujimoto, I.; Tanaka, F.; Nakagawa, T.; Ikeda, T.; Menon, K.K.; Hase, S.; Wada, H.; Ikenaka, K. Isolation and characterization of an N-linked oligosaccharide that is significantly increased in sera from patients with non-small cell lung cancer. *J. Biochem.* **2001**, *129*, 537-542.
8. Ishii, A.; Ikeda, T.; Hitoshi, S.; Fujimoto, I.; Torii, T.; Sakuma, K.; Nakakita, S.; Hase, S.; Ikenaka, K. Developmental changes in the expression of glycogenes and the content of N-glycans in the mouse cerebral cortex. *Glycobiology* **2007**, *17*, 261-276.
9. Yoshimura, T.; Hayashi, A.; Handa-Narumi, M.; Yagi, H.; Ohno, N.; Koike, T.; Yamaguchi, Y.; Uchimura, K.; Kadomatsu, K.; Sedzik, J.; Kitamura, K.; Kato, K.; Trapp, B.D.; Baba, H.; Ikenaka, K. GlcNAc6ST-1 regulates sulfation of N-glycans and myelination in the peripheral nervous system. *Sci. Rep.* **2017**, *7*, 42257.

Supplementary Table S1. Reproducibility of *N*-glycan recovery rate for 3 *N*-glycans.

Pak No.	4	13	15
Area (mean ± SD)	23.92 ± 1.5	14.72 ± 0.8	17.87 ± 1.1
CV value (%)	6.2	5.7	6.1

Supplementary Table S2. ROC curve of A2G1(6)FB in HCC.

	HCV-HCC/LC
Area	0.9614
Std. Error	0.0346
95% confidence interval	0.8936 to 1.029
P value	<0.0001

Supplementary Table S3. Sensitivity and specificity of A2G1(6)FB in HCC.

			HCV-HCC/HC		
	Sensitivity%	95% CI	Specificity%	95% CI	Likelihood ratio
> 0.02	100	93.51% to 100%	2.817	0.343% to 9.808%	1.029
> 0.045	100	93.51% to 100%	4.225	0.88% to 11.86%	1.044
> 0.055	100	93.51% to 100%	5.634	1.556% to 13.8%	1.06
> 0.065	100	93.51% to 100%	12.68	5.964% to 22.7%	1.145
> 0.075	100	93.51% to 100%	18.31	10.13% to 29.27%	1.224
> 0.085	100	93.51% to 100%	28.17	18.13% to 40.1%	1.392
> 0.095	100	93.51% to 100%	40.85	29.32% to 53.16%	1.69
> 0.105	100	93.51% to 100%	49.3	37.22% to 61.44%	1.972
> 0.115	100	93.51% to 100%	61.97	49.67% to 73.24%	2.63
> 0.125	100	93.51% to 100%	66.2	53.99% to 77%	2.958
> 0.135	100	93.51% to 100%	70.42	58.41% to 80.67%	3.381
> 0.145	100	93.51% to 100%	76.06	64.46% to 85.39%	4.176
> 0.155	100	93.51% to 100%	80.28	69.14% to 88.78%	5.071
> 0.165	100	93.51% to 100%	84.51	73.97% to 92%	6.455
> 0.18	100	93.51% to 100%	91.55	82.51% to 96.84%	11.83
> 0.2	100	93.51% to 100%	95.77	88.14% to 99.12%	23.67
> 0.22	100	93.51% to 100%	97.18	90.19% to 99.66%	35.5
> 0.235	98.18	90.28% to 99.95%	98.59	92.4% to 99.96%	69.71
> 0.245	96.36	87.47% to 99.56%	98.59	92.4% to 99.96%	68.42
> 0.26	94.55	84.88% to 98.86%	100	94.94% to 100%	
> 0.275	92.73	82.41% to 97.98%	100	94.94% to 100%	
> 0.285	90.91	80.05% to 96.98%	100	94.94% to 100%	
> 0.3	87.27	75.52% to 94.73%	100	94.94% to 100%	
> 0.315	85.45	73.34% to 93.5%	100	94.94% to 100%	
> 0.325	80	67.03% to 89.57%	100	94.94% to 100%	
> 0.335	78.18	64.99% to 88.19%	100	94.94% to 100%	
> 0.345	74.55	61% to 85.33%	100	94.94% to 100%	
> 0.355	72.73	59.04% to 83.86%	100	94.94% to 100%	
> 0.365	70.91	57.1% to 82.37%	100	94.94% to 100%	
> 0.375	69.09	55.19% to 80.86%	100	94.94% to 100%	
> 0.385	67.27	53.29% to 79.32%	100	94.94% to 100%	
> 0.395	65.45	51.42% to 77.76%	100	94.94% to 100%	
> 0.405	63.64	49.56% to 76.19%	100	94.94% to 100%	
> 0.415	61.82	47.73% to 74.59%	100	94.94% to 100%	
> 0.43	60	45.91% to 72.98%	100	94.94% to 100%	
> 0.445	56.36	42.32% to 69.7%	100	94.94% to 100%	
> 0.455	54.55	40.55% to 68.03%	100	94.94% to 100%	
> 0.47	52.73	38.8% to 66.35%	100	94.94% to 100%	
> 0.485	49.09	35.35% to 62.93%	100	94.94% to 100%	
> 0.495	47.27	33.65% to 61.2%	100	94.94% to 100%	
> 0.505	40	27.02% to 54.09%	100	94.94% to 100%	
> 0.515	38.18	25.41% to 52.27%	100	94.94% to 100%	
> 0.525	34.55	22.24% to 48.58%	100	94.94% to 100%	
> 0.54	32.73	20.68% to 46.71%	100	94.94% to 100%	
> 0.555	30.91	19.14% to 44.81%	100	94.94% to 100%	
> 0.575	29.09	17.63% to 42.9%	100	94.94% to 100%	
> 0.595	27.27	16.14% to 40.96%	100	94.94% to 100%	
> 0.605	25.45	14.67% to 39%	100	94.94% to 100%	
> 0.615	21.82	11.81% to 35.01%	100	94.94% to 100%	
> 0.645	18.18	9.079% to 30.9%	100	94.94% to 100%	
> 0.675	16.36	7.766% to 28.8%	100	94.94% to 100%	
> 0.69	14.55	6.495% to 26.66%	100	94.94% to 100%	
> 0.72	12.73	5.274% to 24.48%	100	94.94% to 100%	
> 0.75	10.91	4.11% to 22.25%	100	94.94% to 100%	
> 0.765	9.091	3.018% to 19.95%	100	94.94% to 100%	
> 0.785	7.273	2.017% to 17.59%	100	94.94% to 100%	
> 0.805	5.455	1.139% to 15.12%	100	94.94% to 100%	
> 0.88	1.818	0.04602% to 9.719%	100	94.94% to 100%	

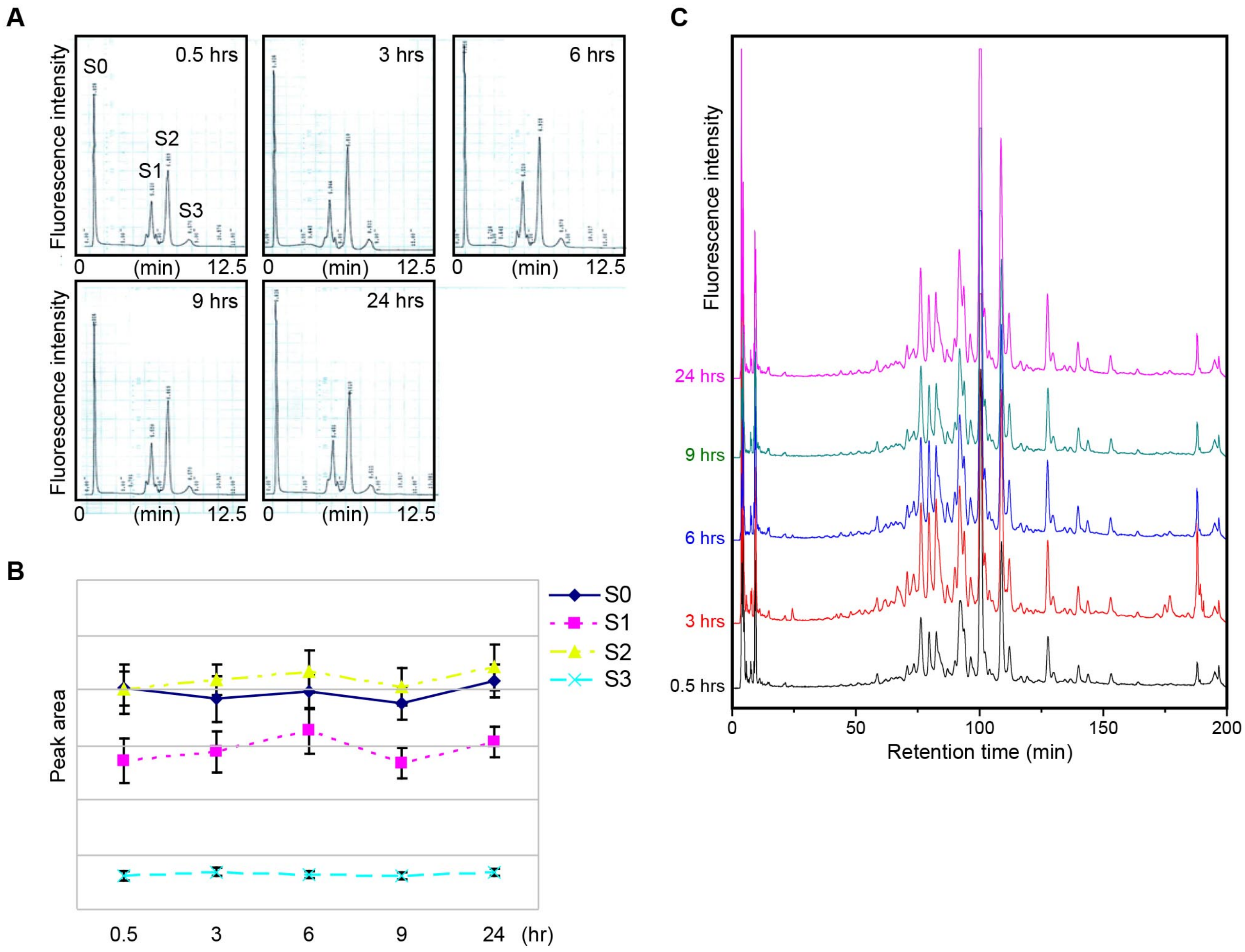

Supplementary Figure S1. Stability of sialylated *N*-glycans in sera.

(A) Chromatogram of anion-exchange chromatography showing the separation of *N*-glycans according to the number of sialic acid residues. S0, S1, S2 and S3 indicate the elution positions of neutral, monosialo, disialo and trisialo PA-oligosaccharides, respectively. (B) Levels of sialylated *N*-glycans in sera prepared at 0.5 h, 3 h, 6 h, 9 h, and 24 h after blood collection. No changes in the level of sialylated *N*-glycans were observed. Error bars represent standard deviation (SD). (C) Chromatogram of NP-HPLC after neuraminidase treatment. *N*-glycan elution profiles were stable 24 h after blood collection.

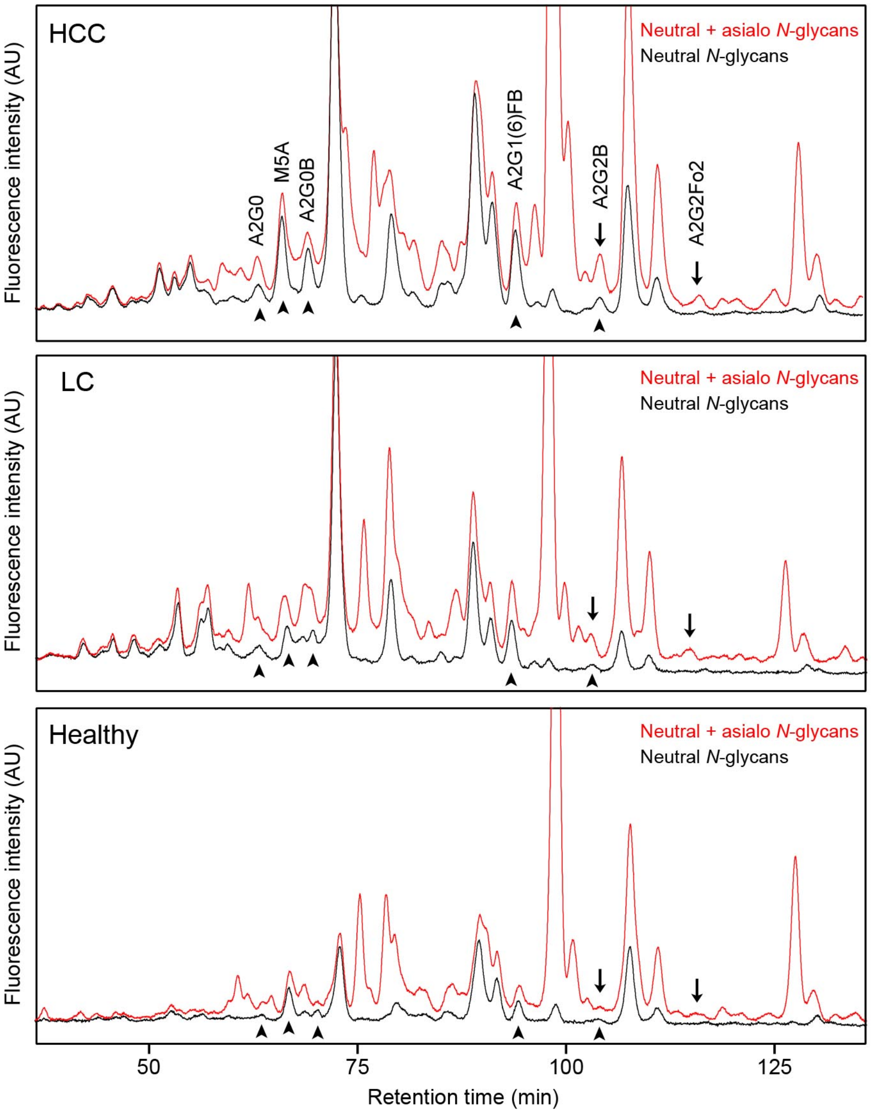

Supplementary Figure S2. NP-HPLC chromatogram of neutral and neutral + asialo *N*-glycans.

Distinct changes of neutral *N*-glycans in HCC and LC patients compared with those in healthy controls. Arrowheads and arrows indicate neutral *N*-glycans and neutral + asialo *N*-glycans respectively. *HCC: a patient with hepatocellular carcinoma, LC: a patient with liver cirrhosis, Healthy: healthy control*.

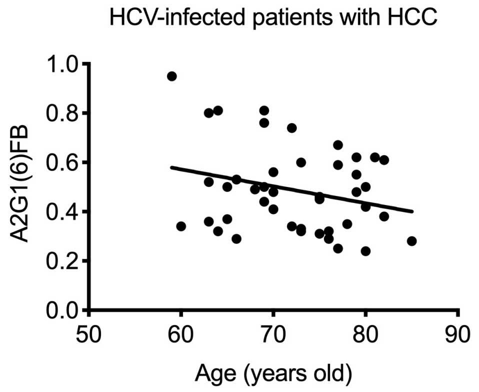

Supplementary Figure S3. Correlation between age and A2G1(6)FB expression in HCV-infected HCC patients. There was no significant correlation between age and A2G1(6)FB expression (r = -0.2635, P = 0.0878) (Pearson's r).

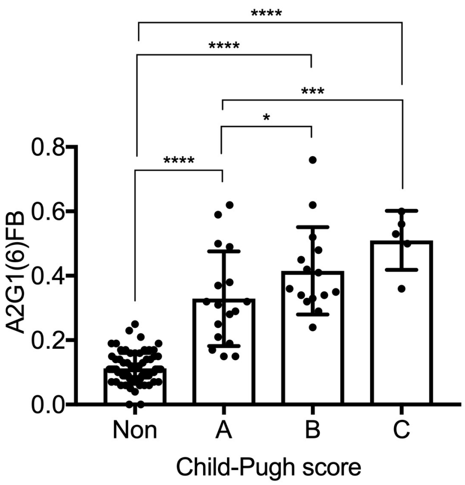

Supplementary Figure S4. A2G1(6)FB expression levels and Child-Pugh classification.

A2G1(6)FB expression levels were examined in groups classified by the Child-Pugh score (Non (n=71) vs A (n=17); P<0.0001, Non (n=71) vs B (n=15); P<0.0001, Non (n=71) vs C (n=5); P<0.0001, A (n=17) vs B (n=15); P=0.0316, A (n=17) vs C (n=5); P=0.0005) (one-way ANOVA). *Non: non-classified samples.*

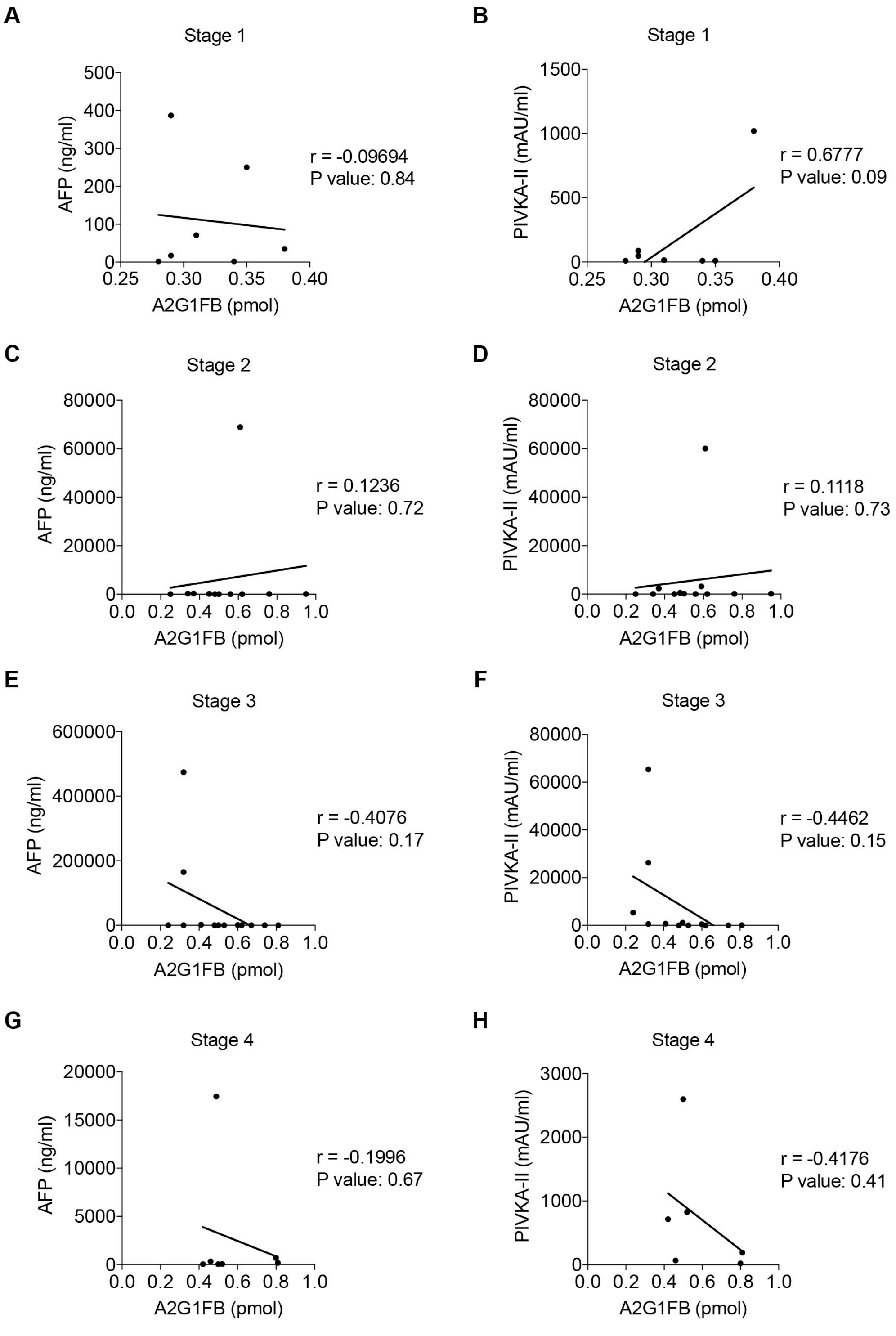

Supplementary Figure S5. A2G1(6)FB expression level is not correlated with AFP or PIVKA-II.

(A–H) Correlations in each stage of hepatocellular carcinoma between A2G1(6)FB and AFP or PIVKA-II. There were no correlations in all stages of HCC. *AFP: α-fetoprotein, PIVKA-II: protein induced by vitamin K absence or antagonists-II.*
